# Supplementary material for: Src Inhibition Attenuates Liver Fibrosis by Preventing Hepatic Stellate Cell Activation and Decreasing Connective Tissue Growth Factor
Source: Cells. 2020 Feb 27;9(3):558. doi: 10.3390/cells9030558 (PMC7140470; doi:10.3390/cells9030558)
Supplement: Supplementary file 1 [file cells-09-00558-s001.pdf]

Western blot analysis

anti-phospho-c-Jun N-terminal kinase (JNK; Thr182/Tyr185)(cs-9251), anti-JNK(cs-9252), anti-phospho-extracellular signal-regulated kinase (ERK; Th202/Tyr204)(cs-4370), anti-ERK (cs-9102), anti-phospho-STAT3 (Tyr705)(cs-9138), anti-STAT3 (cs-4904), anti-phospho-EGFR(Tyr1068)(cs-2234) and anti-EGFR(cs-2232) were purchased from Cell Signaling Technology (Beverly, MA, USA).

Gene	Forward	Reverse
Src	5’- GAACCTGGTGTGCAAAGTGG -3’	5’- TGAGCTCGGTCAGCAGAATC -3’
Fyn	5’- CTTTGGGGGTGTGAACTCCT -3’	5’- TTCTGCCTGGATGGAGTCAA -3’
Lyn	5’- AGCTCCAGAGGCCATCAACT -3’	5’- CACATCTGCGTTGGTTCTCC -3’
Yes	5’- TTGAAGGAAAGCTTCTGGCA -3’	5’- CCAGACCTTGAGTCTGGGGT -3’
Collagne type I	5’-GCCTTGGAGGAAACTTTGCTT-3’	5’-GCACGGAAACTCCAGCTGAT-3’
αSMA	5’-CAGGCTGTGCTGTCCCTCTA-3’	5’-CGGCAGTAGTCACGAAGGAA-3’
CTGF	5’-CCAGACCCAACTATGATGCG-3’	5’-GTGTCCGGATGCACTTTTTG-3’
PAI-1	5’-AAATCCCACACAGCCCATCA-3’	5’-GGACCACCTGCTGAAACACTTT-3’
GAPDH	5’- ACGACCCCTTCATTGACCTC-3’	5’-ATGATGACCCTTTTGGCTCC-3’
TGF-β	5’-AAATCAACGGGATCAGCCCC-3’	5’-GGATCCACTTCCAACCCAGG-3’

Supplementary Table S1. Real-Time RT-PCR primer sequences

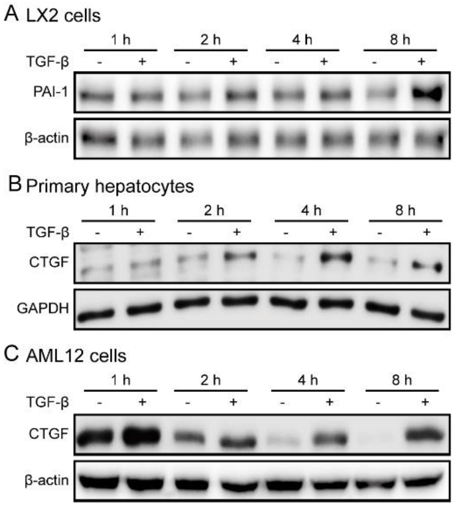

Supplementary Figure S1 LX2 cells, primary hepatocytes, and AML12 cells were treated with 5 ng/mL TGF-β for the indicated durations. Expression of CTGF and PAI-1 was investigated by western blot analysis

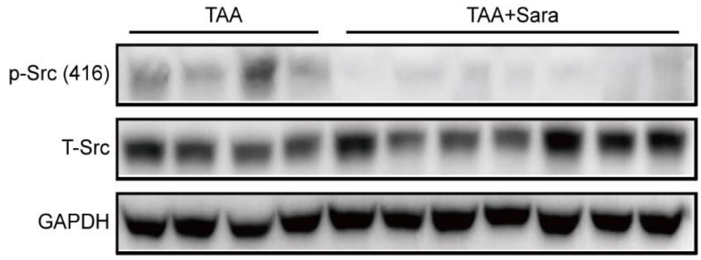

Supplementary Figure S2. Representative western blot analysis of phospho-Src in liver tissues of TAA-injected mice treated with or without saracatinib

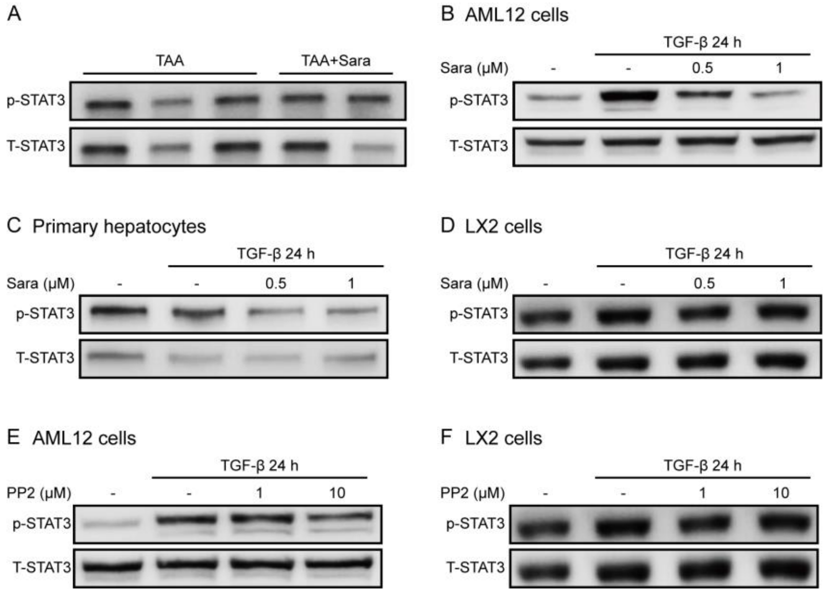

Supplementary Figure S3. Effects of saracatinib and PP2 on STAT3 phosphorylation in liver tissues of TAA-injected mice and TGF-β-treated cells. (**A**) Representative western blot analysis of phospho-STAT3 in liver tissues of TAA-injected mice treated with or without saracatinib. (**B-D**) Western blot analysis of the effect of saracatinib on TGF-β-induced phospho-STAT3 expression in AML12 cells, primary hepatocytes, and LX2 cells. (**E,F**) Western blot analysis of the effect of PP2 on TGF-β-induced phospho-STAT3 expression in AML12 cells and LX2 cells.

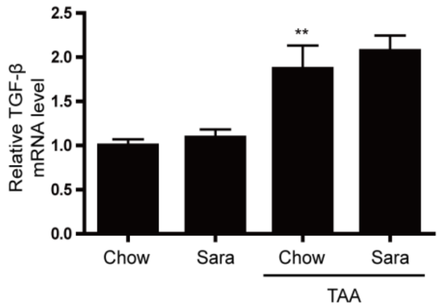

Supplementary Figure S4. Effects of saracatinib on TGF-β mRNA levels in liver tissues of TAA-injected mice. Representative real-time RT-PCR analysis of TGF-β mRNA expression. $^{**}p < 0.01$ compared with compared with the chow group.

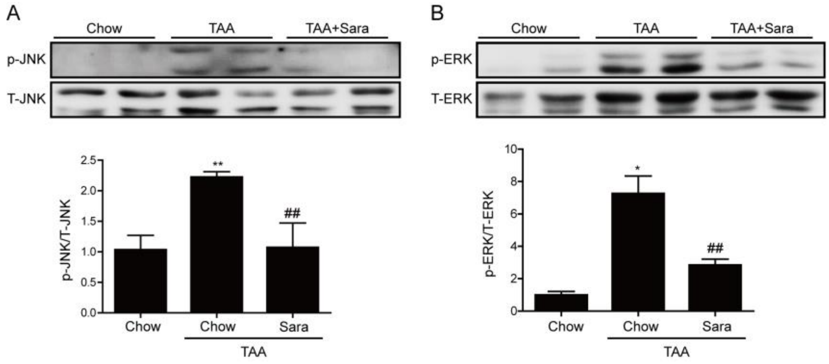

Supplementary Figure S5. Saracatinib attenuates phospho-JNK and phospho-ERK expression in liver tissues of TAA-injected mice. (**A,B**) Representative western blot analysis of phospho-JNK and phospho-ERK in liver tissues of TAA-injected mice treated with or without saracatinib. Data in the bar graphs are means ± SEM. $^{*}p < 0.05$, $^{**}p < 0.01$ compared with control, $^{\#\#}p < 0.01$ compared with the TAA-injected chow group.

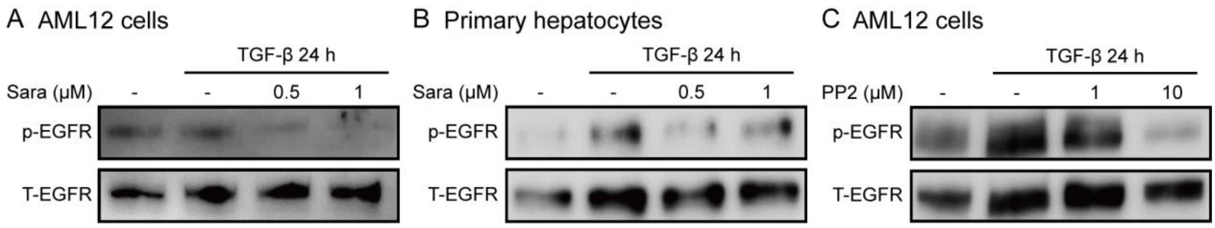

Supplementary Figure S6. Effects of saracatinib and PP2 on TGF-β-induced EGFR phosphorylation. (**A,B**) Western blot analysis of the effect of saracatinib on TGF-β-induced phospho-EGFR expression in AML12 cells and primary hepatocytes. (**C**) Western blot analysis of the effect of PP2 on TGF-β-induced phospho-EGFR expression in AML12 cells.
